# Processed Electroencephalography-Guided Total Intravenous Anesthesia in a Malignant Hyperthermia-Susceptible Child Undergoing Ventricular Septal Defect Repair: A Case Report

**DOI:** 10.7759/cureus.117940

**Published:** 2026-10-14

**Authors:** Adam Was, Maitreyi Narayan, Jonathan Decker, Vikram Sood

**Affiliations:** 1 Anesthesiology, University of Michigan, Ann Arbor, USA; 2 Cardiac Surgery, University of Michigan, Ann Arbor, USA

**Keywords:** cardiopulmonary bypass, congenital cardiac surgery, congenital heart disease, malignant hyperthermia, processed electroencephalography, sedline, total intravenous anesthesia

## Abstract

Malignant hyperthermia (MH) is a rare, life-threatening reaction to certain anesthetic medications that can occur during surgery. Managing an MH-susceptible child for congenital cardiac surgery is particularly challenging: cardiopulmonary bypass (CPB), with its attendant hypothermia and subsequent rewarming, can obscure the early signs of an MH crisis. Total intravenous anesthesia (TIVA) is utilized for MH-susceptible patients, but titrating anesthetic depth during CPB can be complicated, and processed electroencephalography (pEEG) has been studied sparingly in the pediatric cardiac population.

We describe the anesthetic management of a two-year-old, 10.4-kg child with a likely pathogenic variant in the ryanodine receptor 1 (RYR1) gene who underwent ventricular septal defect (VSD) repair with CPB. A trigger-free environment was established, general anesthesia was maintained with a propofol-based TIVA technique, and anesthetic depth was monitored with a SedLine® brain function monitor (Masimo Corporation, Irvine, USA), using the raw electroencephalogram (EEG), spectral edge frequency 95 (SEF95), and density spectral array (DSA). The intraoperative course was uneventful, and no features of MH developed. This case illustrates the feasibility of pEEG-guided TIVA in an MH-susceptible child undergoing congenital cardiac surgery with CPB, and the value of interpreting the spectral display rather than a single index value during hypothermia and rewarming.

## Introduction

Malignant hyperthermia (MH) is an inherited hypermetabolic disorder of skeletal muscle triggered in susceptible individuals by exposure to volatile inhalational anesthetics or the depolarizing muscle relaxant succinylcholine [1]. The underlying defect is dysregulated calcium release from the sarcoplasmic reticulum. Most cases are attributable to a pathogenic variant in the ryanodine receptor 1 (RYR1) gene, which encodes the principal calcium-release channel of skeletal muscle; a small minority involve CACNA1S, which encodes the voltage-gated calcium channel functionally coupled to it [1]. An early diagnostic clue is a rise in end-tidal carbon dioxide (CO_2_) despite increased minute ventilation, typically accompanied by tachycardia and sometimes by muscle rigidity; hyperthermia, acidosis, hyperkalemia, and rhabdomyolysis follow as the crisis progresses, and the syndrome is likely to be fatal if untreated [1]. The reported incidence of MH reactions ranges from approximately one in 10,000 to one in 250,000 anesthetics, and the incidence in children is approximately fivefold higher than in adults [1,2].

For patients known to be MH-susceptible, guidelines uniformly recommend avoidance of triggering agents and delivery of a trigger-free anesthetic, commonly total intravenous anesthesia (TIVA) with propofol, supplemented by opioids and non-depolarizing neuromuscular blockade, or regional techniques where feasible [3]. These principles are considerably harder to apply during congenital cardiac surgery with cardiopulmonary bypass (CPB) for two reasons.

The first concerns temperature. During CPB, patients are commonly cooled deliberately to reduce metabolic demand [4]. An evolving MH crisis declares itself through increased CO_2_ production and heart rate and later temperature; however, the application of CPB to induce intentional cooling and take over gas exchange and circulation may interfere with the usual diagnostic findings [4]. Published cases are consistent with this: during CPB the signs of MH may be obscured and diagnosis delayed, and in a systematic review of reported cases, the development of clinical signs was associated with rewarming, leading the authors to caution against aggressive rewarming in susceptible patients [4,5]. This evidence consists of case reports, however, and whether rewarming itself precipitates MH or instead unmasks a reaction already underway remains uncertain. Reports of MH in the pediatric cardiac surgical setting are correspondingly rare and include both intraoperative crises and a small number of cases of prophylactic management in known- or suspected-susceptible children [4,6,7]. The consequences of a crisis during congenital cardiac repair with CPB can be severe [8].

The second challenge is assessing anesthetic depth. When administering a volatile anesthetic, the end-tidal agent concentration provides a continuous estimate of the dose delivered; during TIVA there is no equivalent measurement, and the pharmacokinetic models used to predict propofol concentrations are less well validated in young children [9]. Processed electroencephalography (pEEG) offers a measure of anesthetic effect on the brain, recorded from electrodes on the forehead. Monitors display the raw electroencephalogram (EEG) waveform; a density spectral array (DSA), which is a color-coded plot of how EEG power is distributed across frequencies over time; and summary values derived from these, including the spectral edge frequency 95 (SEF95), the frequency below which 95% of EEG power is found, which decreases as anesthetic depth increases [10]. An SEF95 range of approximately 6 to 14 Hz has been described as consistent with general anesthesia in children, whereas proprietary numeric indices, whose algorithms were developed largely from adult data, are of uncertain validity in infants and young children [11,12]. During hypothermic CPB, these indices also change with temperature independently of anesthetic dose [13]. This places a premium on interpreting the raw waveform, SEF95, and DSA directly, an approach that has been reported in only a limited number of pediatric studies and less often during hypothermic CPB [14,15].

Here we describe the perioperative anesthetic management of a child with a likely pathogenic RYR1 variant undergoing ventricular septal defect (VSD) repair with CPB, in whom a propofol-based trigger-free technique was combined with continuous SedLine® pEEG monitoring (Masimo Corporation, Irvine, USA). The case addresses a specific clinical question: how anesthetic depth can be titrated reliably during a trigger-free anesthetic in a young child undergoing hypothermic CPB when neither end-tidal agent concentration nor a proprietary pEEG index is dependable. Two recent reports of non-triggering anesthesia for pediatric cardiac surgery with CPB have described infants with a family history of MH rather than genetically established susceptibility; in one, dexmedetomidine was titrated to a bispectral index (BIS) target, and in the other, pEEG monitoring was not used because of the patient's age [16,17]. Consent to publish this report was obtained from the patient's parents.

## Case presentation

Patient and preoperative assessment

A two-year-old boy (weight 10.4 kg, height 83.2 cm, body surface area 0.493 m^2^) with moderate perimembranous VSD and double-chamber right ventricle (DCRV) was scheduled for elective surgical repair. He was born prematurely at 32 weeks' gestation as a twin, and his medical history was also notable for congenital hydrocephalus status post ventriculoperitoneal (VP) shunt placement and inguinal hernia status post repair. He was scheduled for VSD repair due to the new development of DCRV. His outpatient medications included budesonide-formoterol 80-4.5 mcg/actuation twice daily, as well as albuterol 2.5 mg nebulized solution and oral prednisolone 10 mg daily as needed for asthma exacerbations. He was asymptomatic at the time of repair.

MH susceptibility was identified incidentally on whole genome sequencing performed shortly after birth, which had been obtained because of abnormal findings on newborn physical examination. Sequencing revealed a variant in the RYR1 gene, classified by the evaluating genetic counselor as likely pathogenic and conferring susceptibility to MH; no other clinically significant findings were identified. Quantitative estimates of MH risk were not available for this specific variant, so perioperative management followed its classification. There was no known family history of MH, no prior MH episode, and no associated clinical myopathy or musculoskeletal disease. The patient had undergone six prior non-triggering anesthetics without complications.

Preoperative laboratory studies, echocardiography findings, electrocardiogram (ECG), and radiographs were reviewed. Blood tests showed a mild, clinically insignificant thrombocytosis but were otherwise within normal range, as displayed in Table 1. Chest radiograph redemonstrated the left-sided VP shunt (Figure 1). The ECG showed normal sinus rhythm (Figure 2). Notable echocardiography findings for the operative repair included the perimembranous VSD and DCRV, as shown in Figures 3-4. Additional echocardiography findings not pictured below included trivial tricuspid valve regurgitation, bilateral superior venae cavae, a small patent foramen ovale (PFO), and normal biventricular function.

**Table 1:** Preoperative serum laboratory results. CO_2_: carbon dioxide

Parameter	Normal range	Patient's result
White blood cell count (K/uL)	6.0-15.0	8.3
Hemoglobin (g/dL)	11-14	11.4
Hematocrit (%)	34-41	36.1
Platelet count (K/uL)	150-400	407
Sodium (mmol/L)	132-145	139
Potassium (mmol/L)	3.3-5.1	4.2
Chloride (mmol/L)	98-108	103
CO_2_ (mmol/L)	18-28	24
Urea nitrogen (mg/dL)	5-20	14
Creatinine (mg/dL)	0.2-0.6	0.36
Glucose (mg/dL)	70-180	108
Calcium (mg/dL)	8.4-10.4	10.1

**Figure 1:**
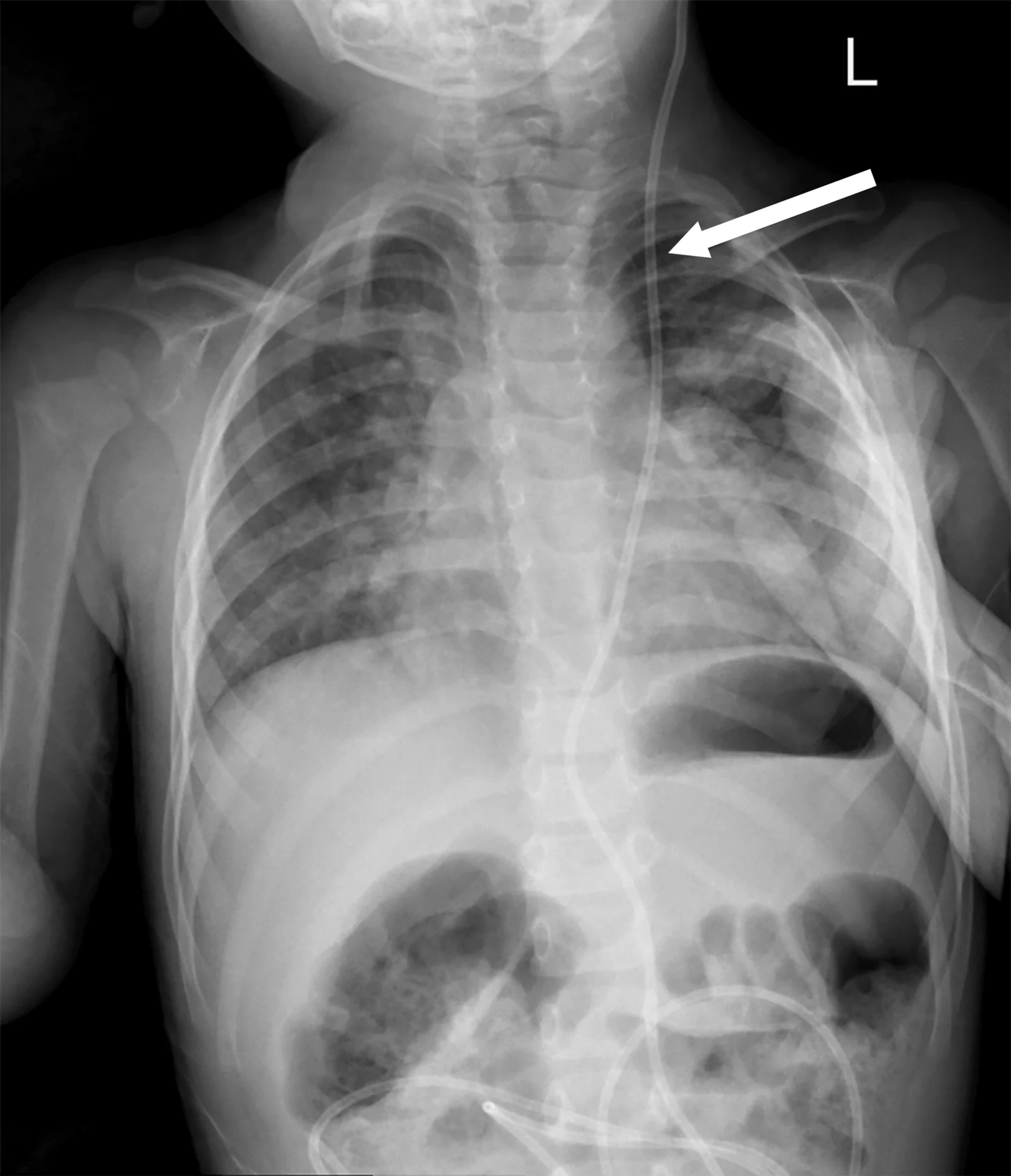
Preoperative chest X-ray with left-sided ventriculoperitoneal shunt tubing (white arrow).

**Figure 2:**
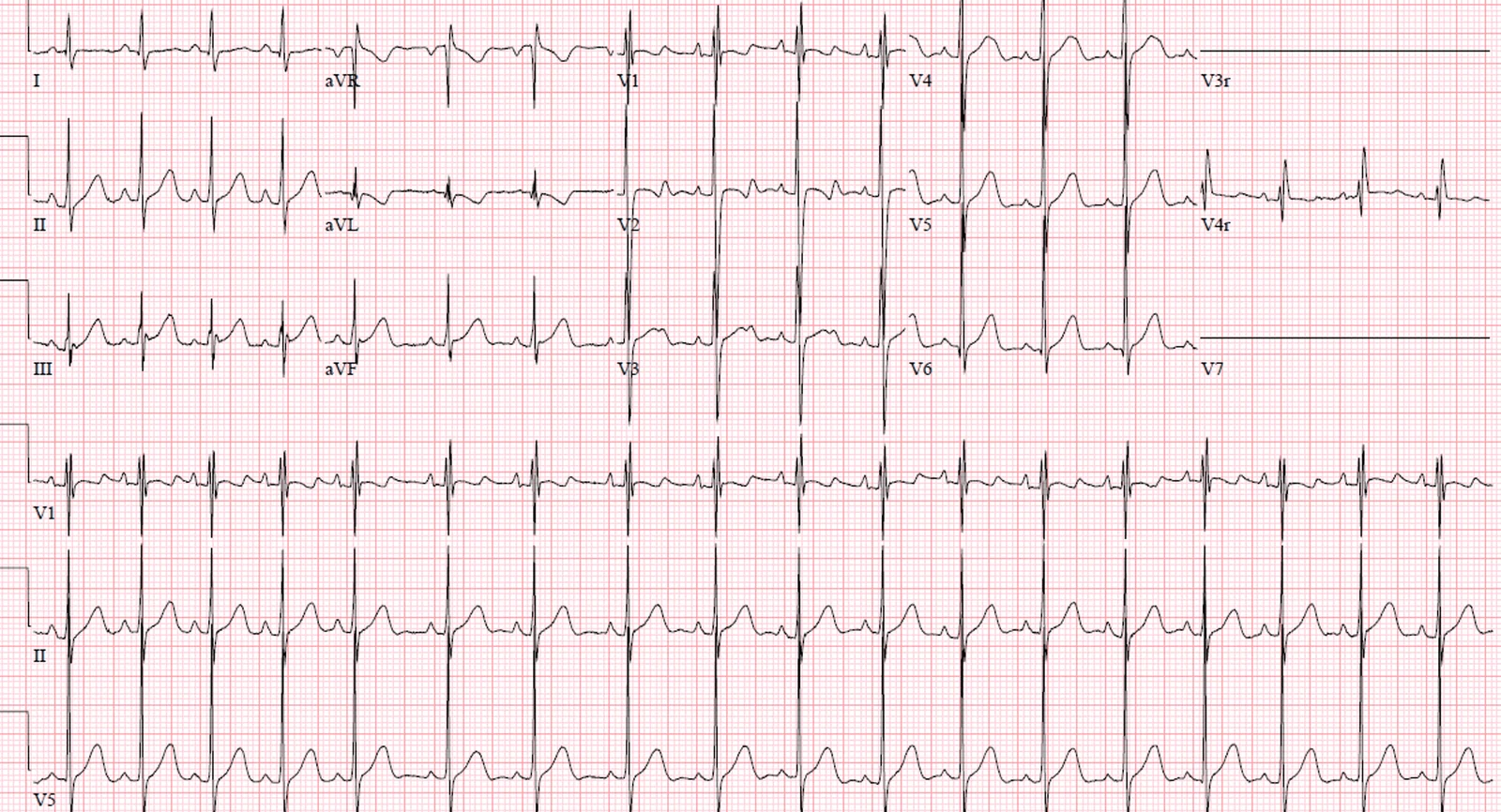
Preoperative electrocardiogram (ECG).

**Figure 3:**
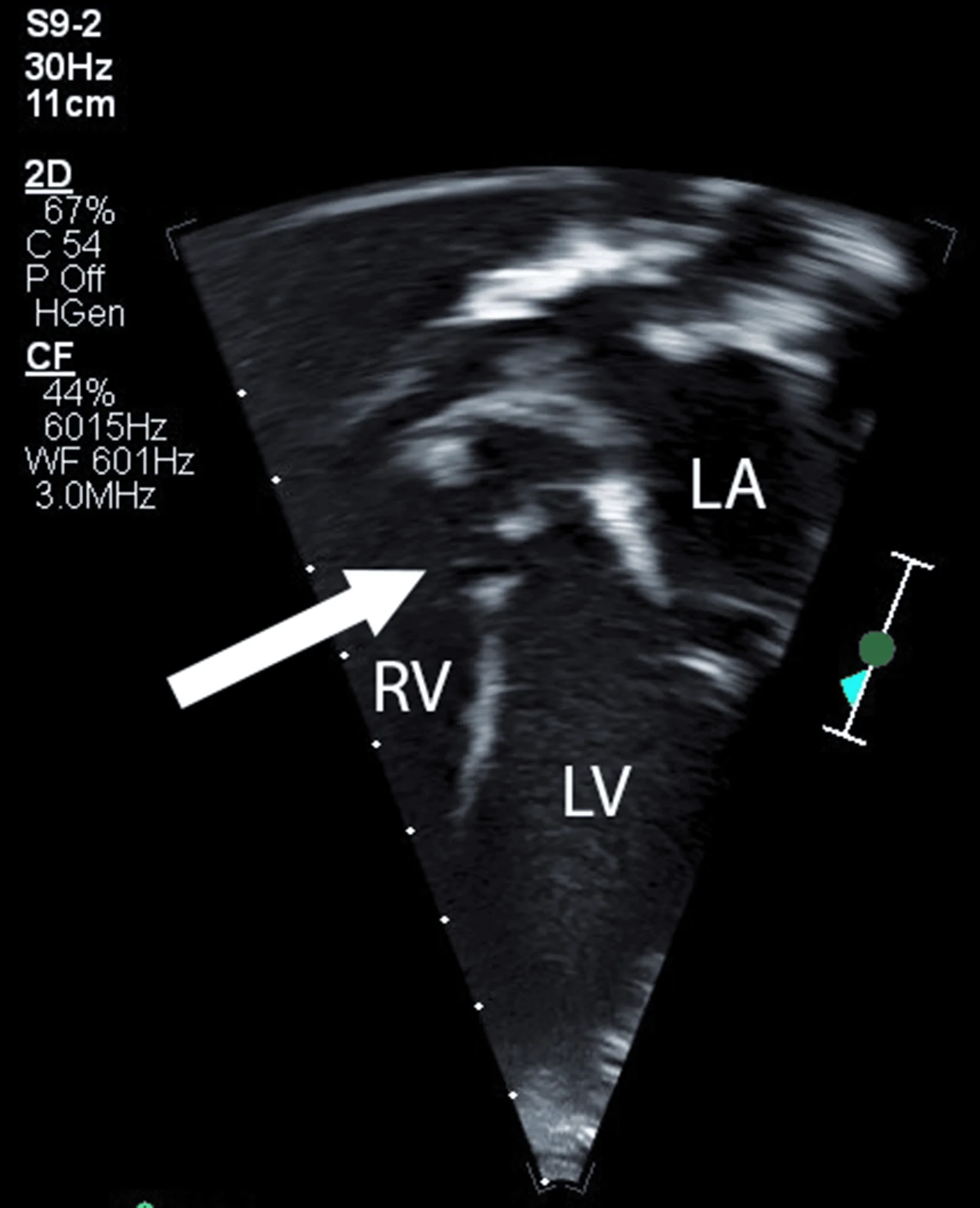
Preoperative transthoracic echocardiogram (apical five-chamber view) showing ventricular septal defect (white arrow). LA: left atrium; LV: left ventricle; RV: right ventricle

**Figure 4:**
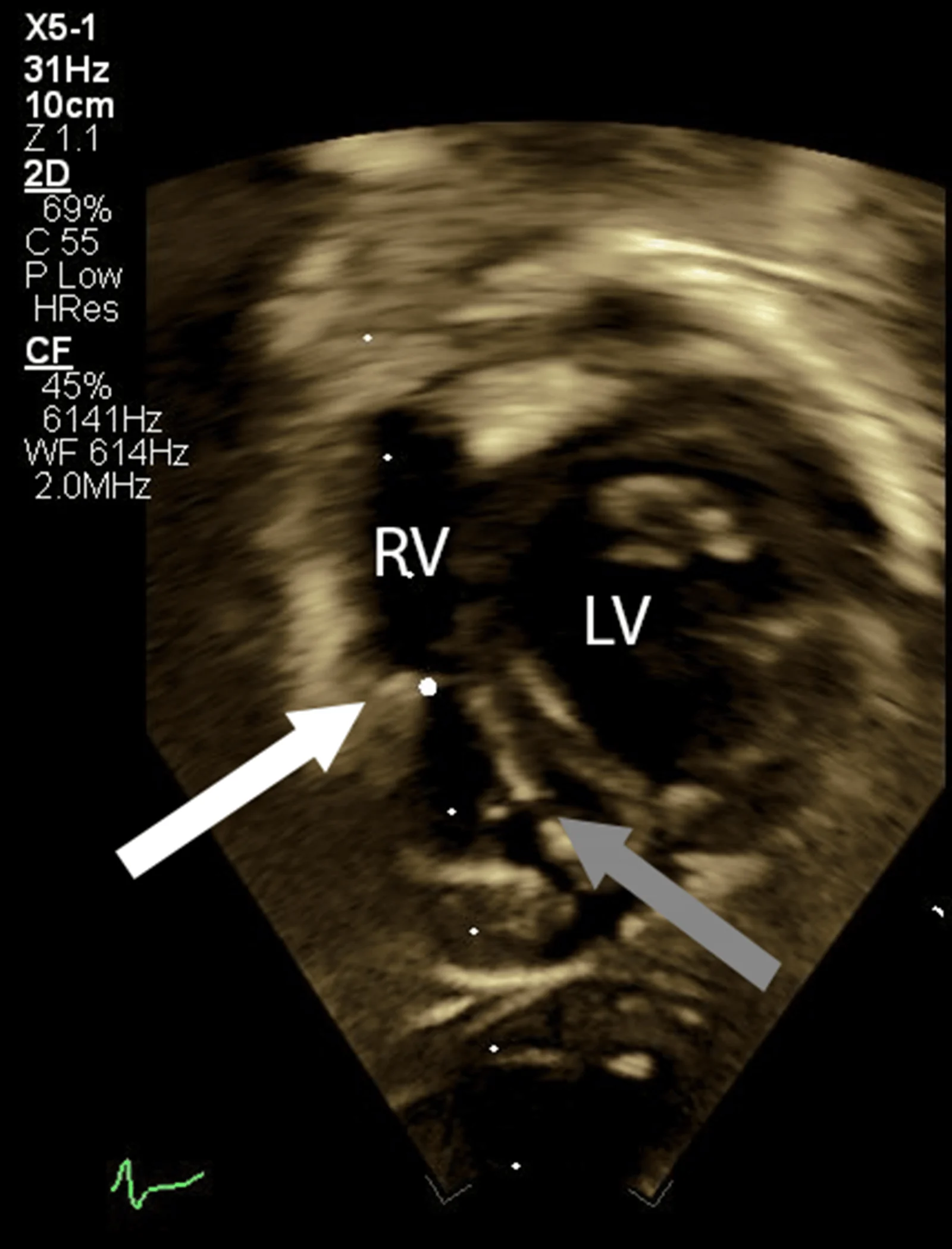
Preoperative transthoracic echocardiogram, subcostal view, showing double-chamber right ventricle (white arrow) and ventricular septal defect (gray arrow). LV: left ventricle; RV: right ventricle

A non-triggering care plan was formulated in coordination with the surgical, perfusion, and nursing teams, including a thorough discussion of the patient’s diagnoses, temperature management protocols throughout the operation, and crisis resource management plans in the event of an MH episode.

Operating room and anesthesia machine preparation

To establish a trigger-free environment, the anesthesia workstation was prepared according to institutional and Malignant Hyperthermia Association of the United States (MHAUS) recommendations [18]. The sevoflurane vaporizer was removed from the anesthesia machine (Datex-Ohmeda, Inc. (GE HealthCare), Madison, USA), and succinylcholine was isolated from the medication cart. A fresh breathing circuit, reservoir bag, and CO_2_ absorbent canister (GE HealthCare, Madison, USA) were placed on the anesthesia machine. It was flushed with high-flow oxygen at 10 L/min for 45 minutes. Activated charcoal filters (Dynasthetics, LLC, Salt Lake City, USA) were also placed on the inspiratory and expiratory limbs. An MH box containing dantrolene was available in the operating room. The CPB machine was prepared with a fresh circuit, and sevoflurane was removed from the machine.

Monitoring

In addition to standard American Society of Anesthesiologists monitors (Masimo Corporation, Irvine, USA), the following were used: invasive arterial blood pressure via a radial catheter (Cook Incorporated, Bloomington, USA), central venous access via the right internal jugular vein, transesophageal echocardiography, bilateral near-infrared cerebral oximetry, and continuous core temperature monitoring at the nasopharyngeal and rectal sites as per institutional protocol. Depth of anesthesia was monitored continuously with the SedLine® brain function monitor using bilateral pediatric sensors (Covidien LLC, Mansfield, USA) applied to the forehead after skin preparation to minimize impedance; the SEF95, raw EEG, and DSA were displayed throughout. pEEG is not used universally at our institution but is applied frequently when clinically beneficial, as in this case. The display was interpreted continuously by the attending anesthesiologist (AW), who has used intraoperative pEEG for 13 years. Dosing was titrated to a target SEF95 of approximately 8-13 Hz, within the previously described range of 6-14 Hz, with a continuous, non-burst-suppressed DSA [11,12]. The planned response to signs of inadequate depth, such as a sustained rise in SEF95 with loss of low-frequency power, was a propofol bolus and an increase in the infusion rate, after first examining the raw waveform to exclude artifact.

Induction and maintenance

The patient received oral midazolam (10 mg, 1 mg/kg) in the preoperative area. Inhaled nitrous oxide was applied briefly during peripheral intravenous catheter placement; although nitrous oxide can increase pulmonary vascular resistance and depress myocardial contractility, its brief use was judged acceptable because the patient had normal biventricular function and no evidence of pulmonary hypertension or elevated pulmonary vascular resistance, and it was discontinued before induction. General anesthesia was induced with intravenous fentanyl 2 mcg/kg, midazolam 0.1 mg/kg, and propofol 2 mg/kg bolus along with 200 mcg/kg/min infusion. Rocuronium 1 mg/kg was given for non-depolarizing neuromuscular blockade. The trachea was intubated without difficulty. Lines and monitors were placed as per above.

Anesthesia was maintained with a continuous propofol infusion at 100-250 mcg/kg/min, titrated to pEEG targets, together with morphine 30 mcg/kg/hr and dexmedetomidine 1 mcg/kg/hr. Midazolam 0.1 mg/kg was re-dosed prior to initiation of CPB. A total of 7 mcg/kg fentanyl was given throughout the case. The depth of neuromuscular blockade was measured with train-of-four (TOF) stimulation of the facial nerve, and rocuronium was re-dosed as needed to maintain a TOF count of one. Muscle tone was monitored by periodic assessment of jaw tone and chest wall compliance. The patient’s anesthetic depth as measured by pEEG was maintained with SEF95 approximately 8-13 Hz and a continuous, non-burst-suppressed DSA pattern. No volatile agent and no succinylcholine were administered at any point. Figure 5 shows representative images of the SEF95 and DSA during various portions of the procedure.

**Figure 5:**
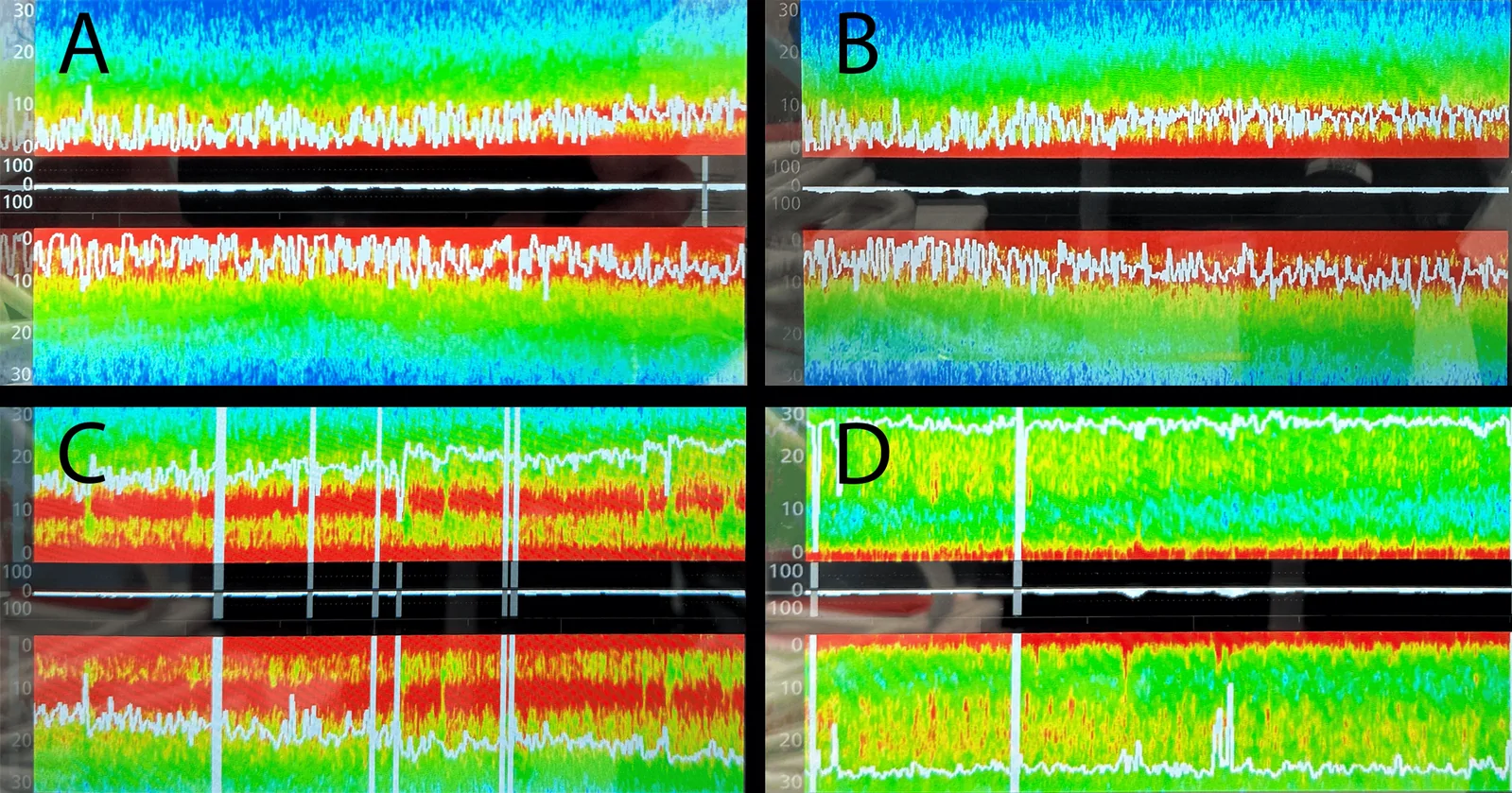
Representative SedLine® display across defined stages of the case (A: maintenance and normothermia; B: hypothermic CPB nadir; C: start of emergence; D: after extubation), showing the SEF95 (white line) and DSA bilaterally. In each panel, the left-hemisphere DSA is displayed above the center and the right-hemisphere DSA below it, as mirror images, with frequency (0-30 Hz) increasing away from the center line, time on the horizontal axis, and EEG power indicated by color (warm colors, higher power; cool colors, lower power). CPB: cardiopulmonary bypass; EEG: electroencephalogram; SEF95: spectral edge frequency 95; DSA: density spectral array

CPB course

CPB was conducted with mild hypothermia to a nadir core temperature of 32.1°C. The patient’s CPB circuit was primed with one unit of cross-matched packed red blood cells. The patient underwent resection of the DCRV via right atriotomy, closure of the perimembranous VSD with a Gore-Tex® patch (W. L. Gore & Associates, Inc., Flagstaff, USA), tricuspid valve repair due to mild central regurgitation noted with intraoperative saline testing, and closure of PFO. CPB time was 88 minutes with an aortic cross-clamp time of 66 minutes.

While administering the propofol infusion, which was continued throughout CPB, the pEEG parameters changed in a manner consistent with the effect of hypothermia: a slight progressive decline in SEF95 and increased lower-frequency power on the DSA during cooling, with recovery on rewarming. These changes were interpreted in the context of temperature rather than as a signal to reduce propofol, and the infusion rate was left unchanged.

Rewarming was performed gradually at a slow rate of no more than 0.5°C every 10 minutes, with careful avoidance of hyperthermia, given the theoretical risk that rewarming could provoke an MH episode in a susceptible patient [19]. Core temperature was not permitted to exceed 37.1°C, and end-tidal CO_2_, arterial blood gases, and temperature were monitored closely for any early features of hypermetabolism, as shown in Table 2. The patient was separated from CPB without difficulty. The patient remained hemodynamically stable, with normal heart rate, oxygenation, and blood pressure, throughout rewarming and termination of CPB.

**Table 2:** Sequential temperature, end-tidal carbon dioxide, and arterial blood gas values. Dashes indicate that end-tidal carbon dioxide (ETCO_2_) was not measured during CPB because the lungs were not ventilated. CPB: cardiopulmonary bypass; ICU: intensive care unit; pCO_2_: partial pressure of carbon dioxide; pO_2_: partial pressure of oxygen; HCO_3_: bicarbonate

Parameter	Normal range	Post-induction, pre-incision	Start of CPB, after cross-clamp placement	On CPB, before cross-clamp removal	After separation from CPB	End of case, on arrival to ICU
Temperature (°C)	36.5-37.5	35.8	34.1	35.5	36.6	36.7
ETCO_2_ (mmHg)	35-45	40	-	-	37	Not measured (patient extubated)
pH	7.35-7.45	7.27	7.31	7.33	7.29	7.33
pCO_2_ (mmHg)	35-45	46	43	44	45	39
pO_2_ (mmHg)	83-108	86	188	211	75	108
Sodium (mmol/L)	132-145	140	139	138	141	146
Potassium (mmol/L)	3.3-5	3.3	4.7	4.5	4.6	4.2
Chloride (mmol/L)	98-108	110	106	106	109	110
Ionized calcium (mmol/L)	1.12-1.30	1.19	1.08	1.17	1.16	1.22
Glucose (mg/dL)	70-180	86	98	115	97	96
Lactate (mmol/L)	0.5-2	0.5	0.8	0.7	0.7	1
HCO_3_ (mmol/L)	18-28	21	22	23	22	20
Anion gap (mmol/L)	4-12	9	11	9	10	15
Hemoglobin (g/dL)	11-14	10.4	9.9	9.2	11.4	10.8

Emergence and postoperative course

At the conclusion of surgery, the propofol infusion was discontinued, and the dexmedetomidine and morphine infusions were decreased. Neuromuscular blockade was reversed with sugammadex 4 mg/kg, in accordance with institutional policy due to TOF of one at the conclusion of surgery. The patient was extubated in the operating room on the day of surgery (D0; subsequent days are numbered D1, D2, and so on). There were no postoperative features of MH; per MHAUS guidelines and institutional practice for an uneventful, non-triggering anesthetic, the patient did not undergo any additional testing [18]. The patient received intravenous acetaminophen, ketorolac, and morphine for postoperative analgesia. The postoperative course was unremarkable; the patient was transferred out of the intensive care unit (ICU) on D1 and discharged home on D4 with follow-up appointments with his neurosurgical, developmental, and cardiology specialists.

## Discussion

This case illustrates the feasibility of combining a propofol-based non-triggering anesthetic with continuous processed EEG monitoring in an MH-susceptible child undergoing congenital cardiac surgery with CPB. The relevant literature can be grouped according to which elements of this case each report shares.

Prior reports

The first group comprises MH crises during cardiac surgery with CPB. A systematic review identified 24 reports describing 26 patients, of whom 14 (54%) developed a crisis during or shortly after bypass [4,5]. Pediatric cases in this group describe previously unrecognized susceptibility presenting as an intraoperative or early postoperative crisis, sometimes with severe neurologic injury [6-8]. The learning point from this group is that the signs of MH are easily obscured during bypass and may emerge during rewarming or after separation, which led some authors to advocate slow rewarming. The second group comprises non-triggering anesthesia for congenital cardiac surgery with CPB in potentially susceptible children. Earlier reports, summarized in the systematic review above, include prophylactic management of children aged two and three years undergoing VSD and atrial septal defect (ASD) repair [4]. More recently, two reports have described infants with a family history of MH: Naguib et al. used dexmedetomidine as the primary agent in a five-month-old undergoing repair of a perimembranous VSD and pulmonary stenosis, titrated to a BIS of 40-60, with recorded values of 30-45; Blansky et al. used remimazolam as an adjunct in a six-month-old undergoing atrioventricular canal repair, without pEEG monitoring because of the patient's age [16,17]. These recent reports establish that a trigger-free technique is practicable with CPB, but they concern suspected rather than genetically established susceptibility, and where pEEG was used, dosing was titrated to the proprietary index, without description of how the EEG changed during cooling and rewarming. The third group comprises reports of TIVA titrated to the BIS in adults with genetically confirmed susceptibility undergoing non-cardiac surgery, such as remimazolam-based TIVA in a patient with a pathogenic RYR1 variant [20]. The fourth describes pEEG in pediatric anesthesia generally and, more recently, SedLine-derived spectral edge frequency and burst suppression in young children with congenital heart disease during extracorporeal circulation, although under sevoflurane rather than TIVA [14,15,21].

What this case adds

The case differs from these reports in two respects. First, susceptibility was genetically established rather than inferred from family history, and it was identified incidentally, on genome sequencing performed for unrelated indications. RYR1 is among the genes for which the American College of Medical Genetics and Genomics recommends reporting of secondary findings, and as genomic sequencing becomes more widely used, anesthesiologists are likely to encounter more children whose MH susceptibility is known before any anesthetic exposure and in the absence of a family history [22]. Second, anesthetic depth was titrated to the raw EEG and spectral display rather than to a proprietary index, and the changes in these parameters during cooling and rewarming are described; the rationale is discussed in the next section. The anesthetic itself was conventional. Propofol-based TIVA supplemented by dexmedetomidine and opioids is a standard trigger-free technique, whereas the recent pediatric cardiac reports focused on dexmedetomidine as the primary agent or remimazolam as an adjunct [16,17]. Propofol was chosen over remimazolam because of institutional experience with propofol-based TIVA in congenital cardiac surgery and because its EEG signatures in young children are well characterized, which allowed pEEG to be used as intended [11,12]; remimazolam is a reasonable alternative, but because it is a relatively new agent, pediatric evidence for it remains limited [9].

Interpreting pEEG during hypothermic CPB

Processed EEG monitors resolve the frontal EEG into its component frequencies and display the result in three complementary ways. The raw waveform shows the signal itself and is the only view in which artifact and burst suppression (alternating periods of high-amplitude activity and near-isoelectric silence, which indicate excessive depth) can be recognized directly. The DSA plots EEG power at each frequency over time, and the SEF95 summarizes the spectrum as a single frequency [10]. These are not independent measurements but different views of the same signal, and their value lies in concordance: an apparent change in depth is accepted as real only when the waveform, spectrogram, and SEF95 agree. During propofol anesthesia at adequate depth, children older than approximately one year typically show high power in the slow-delta (below 4 Hz) and alpha (approximately 8-12 Hz) bands, with an SEF95 of roughly 6-14 Hz; at emergence, slow and alpha power diminish and higher-frequency activity returns, with a corresponding rise in SEF95 [11,12]. In a pediatric SedLine series, median SEF95 during maintenance was 10-12 Hz, rising to 17-18 Hz at extubation, consistent with the 8-13 Hz maintained in this patient and the rise at emergence [11,15].

Hypothermic CPB introduces several recognized challenges. Cooling reduces cerebral metabolism and alters the EEG independently of anesthetic dose: in adults undergoing cardiac surgery with CPB, the SedLine patient state index fell by approximately 0.8 points for each degree Celsius of cooling and rose by 0.7 points for each degree of rewarming, and the suppression ratio rose during cooling and fell during rewarming, whereas changes in SEF were not related to temperature [13]. The median temperature at the end of cooling in that series, 32.1°C, matched the nadir in this patient [13]. Hemodilution, changes in cerebral perfusion, and electrocautery and other artifacts further alter the signal. These findings support relying on the SEF95 and DSA rather than the index during temperature change. In this patient, the modest decline in SEF95 and increase in low-frequency power during cooling reversed on rewarming while the propofol infusion rate was unchanged, and we therefore interpreted it as a temperature effect rather than as a signal to reduce the infusion; reducing propofol during cooling would risk inadequate depth during rewarming. Because both BIS and SedLine monitors display the SEF95 and a spectrogram, the approach described here is not specific to one platform. However, SEF95 values obtained simultaneously from the two devices in children agree only moderately, with limits of agreement of approximately -4 Hz to +5 Hz, so numeric targets should not be transferred directly between devices [23].

Because both hypothermia of CPB and subsequent rewarming intersect directly with MH pathophysiology, temperature management deserves explicit emphasis. We rewarmed more slowly than usual, capped core temperature at 37.1°C, and maintained continuous multi-site temperature monitoring with a low threshold to investigate any unexplained hypercarbia or tachycardia, consistent with the cautious approach described previously [19].

Limitations

This is a single case using a single pEEG platform. It demonstrates feasibility in one patient rather than the safety or efficacy of the approach, and the absence of MH cannot be attributed solely to the technique, since MH does not occur with every exposure even in susceptible individuals [1]. Generalizability is further limited by the need for pEEG equipment and interpretive expertise, which add cost and are not available in all centers, and by the limited validation of pEEG parameters in young children, particularly during hypothermic CPB. The interpretive observations reported here are descriptive and derived from a single patient.

## Conclusions

An MH-susceptible child underwent VSD repair with CPB using a propofol-based non-triggering anesthetic and continuous processed EEG monitoring, without any features of MH. This single case suggests that pEEG can be incorporated into trigger-free TIVA for congenital cardiac surgery, provided the raw EEG, spectral edge frequency, and DSA are interpreted rather than a proprietary index alone. This is particularly relevant during the hypothermia and rewarming phases of CPB, when both the anesthetic depth signal and the risk profile of an MH-susceptible patient are most dynamic. The practical takeaway is that during cooling and rewarming, changes in the spectral display should be interpreted against core temperature. Feasibility in one patient does not establish safety or efficacy, and wider experience is needed before the approach can be generalized.
